# The potential of CO_2_-based production cycles in biotechnology to fight the climate crisis

**DOI:** 10.1038/s41467-023-42790-6

**Published:** 2023-11-01

**Authors:** Simone Bachleitner, Özge Ata, Diethard Mattanovich

**Affiliations:** 1 University of Natural Resources and Life Sciences, Department of Biotechnology, Institute of Microbiology and Microbial Biotechnology, Vienna, 1190 Austria; 2 Austrian Centre of Industrial Biotechnology, Vienna, 1190 Austria

**Keywords:** Metabolic engineering, Industrial microbiology, Applied microbiology, Synthetic biology

## Abstract

Rising CO_2_ emissions have pushed scientists to develop new technologies for a more sustainable bio-based economy. Microbial conversion of CO_2_ and CO_2_-derived carbon substrates into valuable compounds can contribute to carbon neutrality and sustainability. Here, we discuss the potential of C1 carbon sources as raw materials to produce energy, materials, and food and feed using microbial cell factories. We provide an overview of potential microbes, natural and synthetic C1 utilization pathways, and compare their metabolic driving forces. Finally, we sketch a future in which C1 substrates replace traditional feedstocks and we evaluate the costs associated with such an endeavor.

## Abstract (web summary)

The utilization of one-carbon assimilation pathways for bioproduction represents a promising direction towards a more sustainable bio-based economy. Here, the authors compare the thermodynamic efficiencies and energy demand of C1-assimilation pathways and discuss their implementation for energy, material, and food production.

## Introduction

Fossil resources such as coal, oil, and gas have led to the rapid growth of industrial operations, transportation, and agriculture. Nevertheless, fossil resources are the primary source of direct carbon dioxide (CO_2_) emissions, hence contributing greatly to the climate crisis^1,2^. Although humanity is aware of the risks of using fossil fuels as resources, still about two-thirds of the anthropogenic CO_2_ emissions derive from combusting coal, oil, and gas to gain energy^2^. And notably, of all carbon-based materials, 47% of the annual production is fossil-based^3^. The way we currently produce and utilize materials severely affects our planet and leads to serious environmental issues. Thus, our mission is clear: We need to achieve rapid decarbonization across all sectors and thrive for cleaner production cycles of fuels, chemicals, food, and feed.

Industrial biotechnology represents one promising technology that can save energy and significantly reduce CO_2_ emissions when based on sustainable and renewable resources. Microbial conversion of organic substrates allows the production of biofuels and high- and low-value chemicals^4–6^ and thus, replaces fossil feedstocks as raw materials. Among prospective industrial production cells mainly bacteria and yeasts are being proposed as chassis cells for upcoming synthetic biology applications as they can use various feedstocks from a wide range of sources. The so-called first-generation feedstocks, which mostly consist of sugar and starch, are derived directly from agricultural output^7,8^. Second-generation feedstocks are leftovers from the processing of agricultural raw materials, such as glycerol produced during the generation of biodiesel or lignocellulosic sugars generated from straw or maize stover^8,9^. Notably, these materials are still made from agricultural products and compete with those that are used to make food, animal feed, and plant fibers. Additionally, the lack of available land questions the sustainability of first-generation feedstocks at one point. According to FAO statistics^10^, about one-third of the arable land (38% of the world’s land area) is cultivated while the remaining two-thirds include meadows and pastures for livestock. As the population grows, so does the need for agricultural land. The transition of arable regions to non-agricultural lands, intensive use of pesticides, shorter fallow seasons, and climate change further reduce the amount of land suitable for agriculture^11^.

One alternative to first-generation feedstocks is the valorization of CO_2_ and its chemical conversion to single carbon (C1) substrates as feedstocks. C1 feedstocks have gained significant momentum in recent years^12,13^ as they offer solutions to several issues: First, they can be produced from CO_2_ and thus reduce the amount of free CO_2_ in the atmosphere. Second, their production does not destroy existing chemical structure (as does the hydrolysis of polysaccharides) and third, microbial C1 utilization creates structure entirely from scratch and forms a final product by creating all carbon-carbon bonds. The most promising C1 carbon sources include methanol (MeOH), formate, methane (CH_4_), and carbon monoxide (CO)^12^. Formate, MeOH, and CO can be generated electrochemically from CO_2_ and renewable energy^14,15^. Although electrochemical reduction processes are not yet competitive with fossil resources, technological advancements (e.g. for catalysts) and legislative adjustments, if enacted as currently anticipated, would enable the commercialization of CO_2_ reduction processes in the near future. For further aspects and recent developments on the electrochemical reduction of CO_2_ please refer to the following refs. ^16–18^.

To be able to make an impact on the carbon balance and mitigate the atmospheric CO_2_ levels, two important questions need to be addressed: (i) Which products can we produce from C1 substrates that significantly reduce the carbon footprint, and (ii) which production cycles need to be implemented to realize a C1-based bioeconomy for the future? In the following sections, we give an overview of C1-assimilation pathways that are exploited and compare their thermodynamic efficiencies as well as their energy demand in the form of ATP and NAD(P)H. Further, we provide an opinion on how C1 substrates can be implemented for energy, material, and food production in the future.

## C1-assimilation pathways

Nature offers a great diversity of carbon utilization pathways and so far, seven natural autotrophic carbon-fixation pathways have been characterized. Those include the Calvin-Benson-Bassham (CBB) cycle, the reductive tricarboxylic acid cycle (rTCA), the oxygen-sensitive Wood-Ljungdahl pathway, the 3-hydroxypropionate (3-HP), the hydroxypropionate/4-hydroxybutyrate (HP/HB), and the dicarboxylate/4-hydroxybutyrate (DC/HB) cycles, and the reductive glycine pathway. These are reviewed and compared extensively elsewhere^19–21^.

**Fig. 1:**
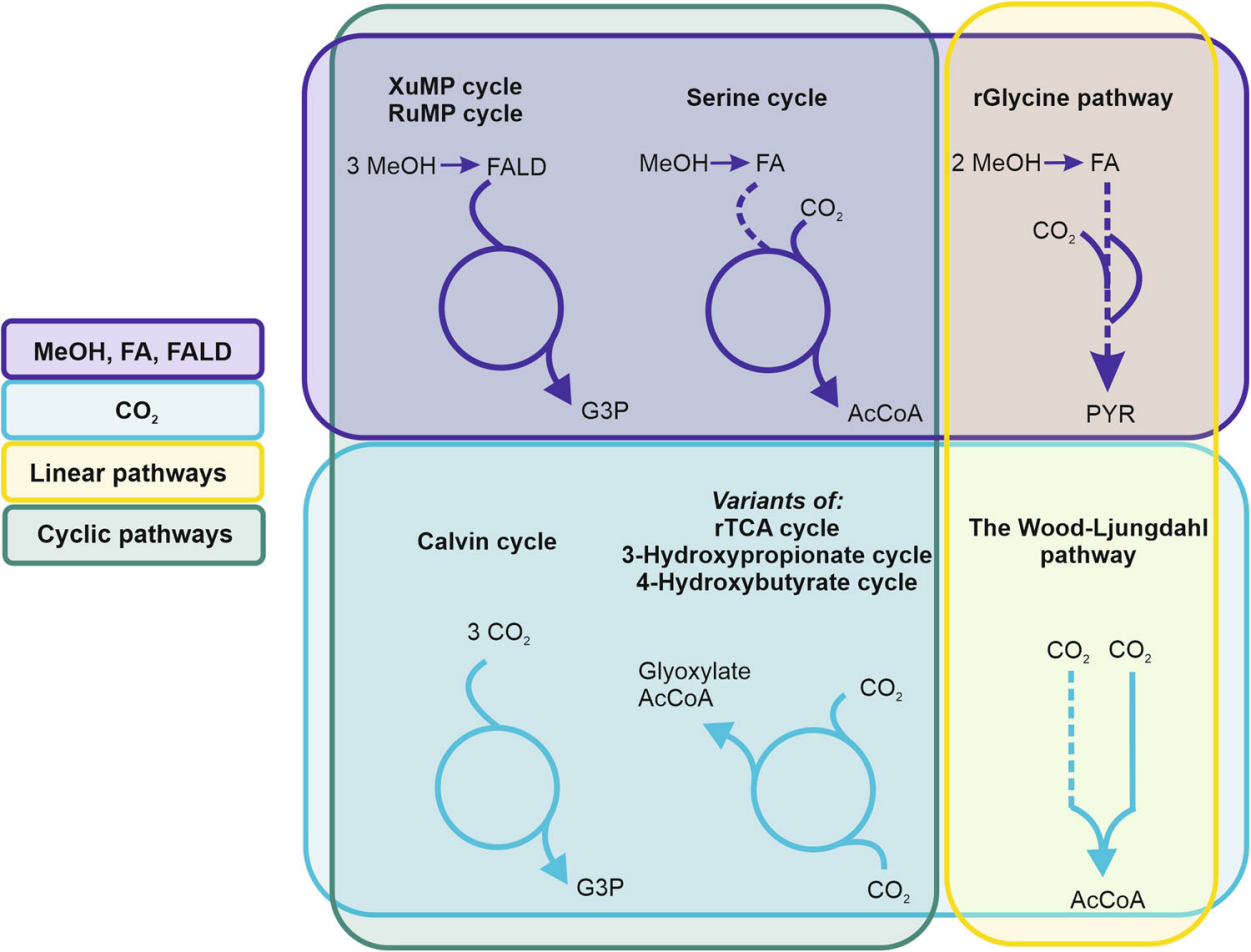
Natural carbon fixation pathways. XuMP Xylulose monophosphate, RuMP Ribulose monophosphate, G3P glyceraldehyde 3-phosphate, AcCoA acetyl CoA, PYR pyruvate, MeOH methanol, FA formate, FALD formaldehyde. Purple: methanol, formate, or formaldehyde fixing pathways, Blue: CO_2_- fixing pathways, Yellow: linear pathways, Green: cyclic pathways. Note that the serine cycle and rGlycine pathway are mixed pathways that incorporate CO_2_ in addition to methanol and formate.

The first step towards a C1-based bioprocess is to select or design an effective microbial pathway for carbon fixation (Fig. 1). Several options exist depending on the used microbial platform, the product of interest, and the parameters of cultivation. In general, autotrophic microorganisms would be an obvious choice for a C1-based production platform as they can grow solely on CO_2_ and an energy source (either from light or chemical reactions)^19^. The CBB cycle is the most abundant pathway to fix CO_2_, used by photoautotrophs and some chemoautotrophs, whereby the latter employ more efficient pathways for energy generation^22^. However, due to their complex nutrient requirements, process demands and difficulties in genome editing, the use of chemoautotrophs is still limited in industrial applications^23^. Phototrophic microorganisms, such as cyanobacteria and eukaryotic microalgae are easier to cultivate and many studies have focused on their utilization in the biotech sector^6,24,25^. Nevertheless, the industrial scale of production is still challenged by technical hurdles in photobioreactors including the light availability, effective distribution, and optimization of wavelength and intensity^22^.

Among autotrophs, acetogens are another choice for a microbial production platform. Acetogenic microorganisms can reduce CO or CO_2_ into acetyl-CoA via the Wood-Ljungdahl pathway, using hydrogen as an energy source. Additionally, some acetogens produce naturally ethanol or acetic acid and are thus used in syngas fermentations (CO_2_, CO, and H_2_) at an industrial scale for the production of biofuels and chemical commodities^26,27^. While waste streams and C1 gaseous substrates can be converted by anaerobic gas fermentation by acetogens, the product spectrum is constrained by their metabolism. Although genetic tools are developed for well-known acetogens, there are still challenges for genome editing which makes metabolic engineering difficult^28^. Recently however, a CO_2_-negative process for the production of acetone and isopropanol from waste gas feedstocks was achieved through sequence mining for superior enzymes and subsequent engineering of *Clostridium autoethanogenum*^29^.

Methylotrophs represent another option as a production platform for the utilization of C1 feedstocks with several advantages. First, methylotrophic microorganisms include several species of bacteria, archaea, and also a few fungi, mostly yeasts^30^. Some of them are well-established in the industry, and knowledge about cultivation, process requirements, and genetic engineering exists^31^. Second, they can utilize formate or MeOH, which are both liquid, fully water soluble, and easily applied substrates and hence regarded attractive feedstocks. And third, aerobic fermentation of MeOH offers a wide product variety, ranging from bulk and fine chemicals to proteins, organic acids, and biomass^32^. Notably, only a few studies have focused on formate as a substrate so far^33,34^.

Additionally, methanotrophs have also acquired popularity as a host for their ability to convert methane into biodiesel or chemical commodities. Methanotrophs can be either aerobic or anaerobic and include species from the proteobacteria phylum but also the archaea domain^35–37^. Previous reviews have focused on aerobic methanotrophs in particular, including environmental detection, occurrence, and prospective uses^38,39^. However, low mass transfer rates and limited methane solubility in aqueous phases provide a difficulty for industrial-scale manufacturing. To overcome such hurdles, process engineering solutions concentrating on reactor configuration and design, or supplementation of exogenous mass transfer vectors, can be used^36^.

The question of whether more efficient novel synthetic routes may be created, together with advancements in synthetic biology, bioinformatics, and biochemistry, led to the design of novel synthetic pathways with possibly faster kinetics^40^. Recently, researchers have been focusing on implementing C1-utilizing pathways in traditional industrial platforms via the introduction of specific metabolic modules^41,42^. Hence synthetic methylotrophy was established in *Escherichia coli, Cornyebacterium glutamicum*, and *Saccharomyces cerevisiae*^43–46^. Similarly, the heterotrophic metabolism of *E. coli* and *Komagataella phaffii* was re-wired to assimilate CO_2_, thus converting them to synthetic autotrophs^47–49^. Further engineering showed the potential of the newly generated autotrophic yeast strains as a production platform for organic acids^50^. Noteworthy, synthetically engineered strains cannot yet compete with natural producer strains. Their metabolite yields are still low and need further metabolic engineering to overcome hurdles in cell energy and carbon utilization efficiency. However, progress in synthetic biology is rapid and might soon generate yeast strains that may serve as a foundation for a production system of chemicals and enzymes from CO_2_ thereby aiding in CO_2_ mitigation in the future.

As mentioned above synthetic biology enables rewiring of native pathways or the design of new networks for specific characteristics. Nature selects not necessarily for efficiency and each pathway has a specific thermodynamic landscape and kinetics and thus comes with certain bottlenecks and challenges to deal with^51^. Hence, in the following section we focus on native carbon fixation pathways that are currently exploited in research for C1- biobased processes, i.e. the CBB cycle, the rTCA cycle, the serine cycle, the reductive glycine pathway, the Wood Ljungdahl pathway as well as the xylulose monophosphate (XuMP) and ribulose monophosphate (RuMP) pathways (Fig. 1 and Table 1). By using eQuilibrator^52^, we calculated their Max-min driving forces (MDF)^53^ and energy requirements in the form of ATP and NAD(P)H (Table 1, detailed data for eQuilibrator in Supplementary Tables 1–9). The MDF is the minimal metabolic driving force, calculated by adjustment of metabolite concentrations by linear optimization to make all pathway reactions as favorable as possible. MDF is expressed as inverse Gibbs free energy (-Δ_r_G’) of the reaction with the lowest numerical value. Pathways with a low MDF have low fluxes and require metabolic engineering with highly abundant enzymes to enable a considerable flux, whereas pathways with a high MDF do not^54^.

**Table 1:** Carbon fixing pathways and their energy demand

Pathway	Net reaction	Input	Output	MDF (kJ/ C-mol)^1^	ATP/ C-mol	NAD(P)H/ C-mol
CBB cycle	5 H_2_O + 9 ATP + 6 NADPH + 3 CO_2_ < = > 6 NADP + 9 ADP + 8 phosphate + glyceraldehyde 3-phosphate	CO_2_	Glyceraldehyde 3-phosphate	2.34	3	2
rTCA cycle	2 ATP + 3 NADH + coenzyme A + 2 CO_2_ + FADH_2_ < = > H_2_O + 2 ADP + 3 NAD + 2 phosphate + acetyl CoA + FAD	CO_2_	Acetyl CoA	0.67^1^ (−13.04)	1	1.5 ( + 0.5 FADH)
Wood Ljungdahl pathway	ATP + coenzyme A + 2 CO_2_ + 4 H_2_ < = > 2 H_2_O + ADP + phosphate + acetyl CoA	CO_2_	Acetyl CoA	0.53^1^ (−8.03)	0.5	2
rGlycine pathway	2 ATP + 2 NADPH + NADH + CO_2_ + 2 formate <=> H_2_O + 2 NADP + 2 ADP + NAD + 2 phosphate + pyruvate	Formate, CO_2_	Pyruvate	1.38	0.67	1
Serine cycle	H_2_O + 3 ATP + NADPH + NADH + coenzyme A + CO_2_+ methanol + ubiquinone <=> NADP + 3 ADP + NAD + 3 phosphate + acetyl CoA + ubiquinol	Methanol, CO_2_	Acetyl CoA	3.50	1.5	1
XuMP cycle	2 H_2_O + 3 ATP + 3 O_2_ + 3 methanol <=> 3 ADP + 2 phosphate + 3 H_2_O_2_ + glyceraldehyde 3-phosphate	Methanol	Glyceraldehyde 3-phosphate	3.59	1	0
XuMP cycle w/o SBP	2 H_2_O + 3 ATP + 3 O_2_ + 3 methanol <=> 3 ADP + 2 phosphate + 3 H_2_O_2_ + glyceraldehyde 3-phosphate	Methanol	Glyceraldehyde 3-phosphate	3.29	1	0
RuMP cycle TA version	ATP + 3 O_2_ + 3 methanol <=> ADP + 3 H_2_O_2_ + glyceraldehyde 3-phosphate	Methanol	Glyceraldehyde 3-phosphate	1.53	0.33	0
RuMP cycle SBP version	H_2_O + 2 ATP + 3 O_2_ + 3 methanol <=> 2 ADP + phosphate + H_2_O_2_+ glyceraldehyde 3-phosphate	Methanol	Glyceraldehyde 3-phosphate	2.01	0.67	0

^1^ In order to achieve a positive max-min driving forces (MDF) value for the rTCA cycle and the Wood Ljungdahl pathway CO_2_ concentrations need to be higher, hence the CO_2_ concentrations were set to 1000 and 10 mM respectively. MDF values with standard CO_2_ concentrations (10 µM) are provided in brackets.

The CBB cycle is the most abundant CO_2_ fixation pathway in nature, however, not necessarily the most efficient metabolic pathway as it requires 3 moles of ATP and 2 moles of NADPH with a mediocre MDF of 2.34 kJ per mole of carbon to assimilate. Noteworthy, this high energy demand enables the CBB cycle to work at very low CO_2_ concentrations. Other CO_2_ fixing pathways such as the rTCA and Wood Ljungdahl pathways require less ATP and reducing power, yet their MDFs are low and need higher CO_2_ concentrations to be thermodynamically feasible (Table 1).

More reduced C1 sources, such as formate and methanol, are metabolized by four distinct pathways: the XuMP, RuMP, rGlycine, and serine cycle, of which the latter two constitute mixed pathways with CO_2_ fixation. Notably, the serine cycle has the highest MDF per C-mol, however requiring high amounts of ATP and NADH. The serine cycle is shortly followed by the XuMP cycle in its MDF value, requiring only 1 ATP per C-mol. Interestingly when we compare the transaldolase variant (TA) with the sedoheptulose-1,7-bisphosphatase (SBP) variant of the RuMP cycle we see significant differences in their MDF and ATP requirements. The RuMP cycle with the TA variant uses half as much ATP, however at a cost of a low MDF, demonstrating that the choice of single enzymes strongly affects the thermodynamic landscape of a pathway. The lowest MDFs are found in the Wood Ljungdahl pathway and the rTCA cycle, ranging between 0.53–0.67 kJ per C-mol, and could only be achieved by increasing the maximum CO_2_ concentration. While the rTCA in anaerobic bacteria involves ferredoxins as electron carriers^55^ we have based the calculation on NADH as ferredoxins are not generally accessible in eQuilibrator due to their wide range of reduction potentials.

Integrating thermodynamic and kinetic models help in engineering more efficient pathways with high MDF and low energy requirements of least thermodynamically favorable reactions. The development of toolboxes, such as *PathParser* or *OptMDFpathway*, identify and facilitate optimization of metabolic pathways for effectiveness and robustness^54,56^. Hence strategies in synthetic pathway designs should include pathway optimization, enzyme kinetics, energy efficiency, favorable thermodynamics, and topological compatibility^40^. ATP and NAD(P)H demand is calculated to the native output metabolites of the respective pathways in Table 1. For overall comparability the demands to the same intermediate metabolite, pyruvate, are provided in Supplementary Table 10.

## Products that can be made from C1 carbon sources

**Fig. 2:**
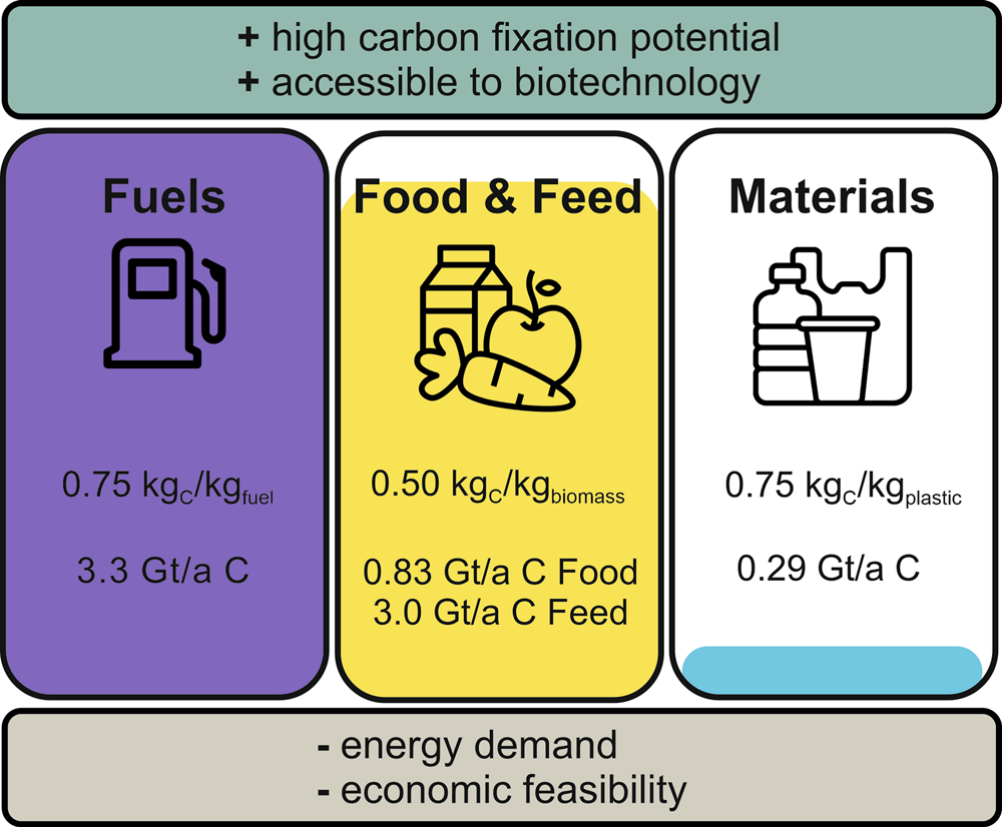
Potential and constraints for C1 feedstocks in the fuel, material, and food and feed sectors. Each sector has a significant carbon fixing potential and is biotechnologically accessible. However, energy consumption and economic feasibility are viewed as hurdles for C1 feedstock implementation, as inexpensive resources have been utilized in all three sectors up to this point. Each sector’s carbon content is provided, indicating the possibility for carbon fixation. For that, the average carbon content was calculated of common fuels (methane, propane, butane, gasoline, diesel, kerosene, heavy fuel oil, ethanol and methanol) and of the six most common plastics (PET, HDPE, PVC, LDPE, PS, PP). In the case of food and feed, a default value for dry biomass was used. Using annual global production values for crude oil^110^, plastics^81^, food^111^ (at the consumers table, without any losses) and feed^112^, an estimation of each carbon content is given for each sector in tons per year carbon (t/a C). Icons for illustrating each sector were provided by Freepik (Fuels), Smahicons (Materials) and FBJan (Food &Feed) at https://www.flaticon.com/.

Synthetic pathway engineering allows C1 utilization in many hosts, and a variety of materials can be produced from a range of microorganisms, using the C1 feedstocks methanol, formate, methane, CO, or CO_2_^12,35,57–59^. Although we see these huge leaps in metabolic engineering and synthetic biology, which provide us with more possibilities to exploit microorganisms for various products, the question remains to which extent C1 feedstocks help us to bind CO_2_ emissions and thus, mitigate climate change. Which products abate CO_2_ emissions the most? And how much energy is necessary to generate C1 carbon feedstock? In the next paragraphs, we have a look at the fuel, material, and food and feed sectors, that are responsible for a majority of the CO_2_ emissions and envisage how C1 carbon sources can or cannot revolutionize these sectors (Fig. 2). However, these possibilities come with the prerequisite to be open to a drastic change and turn away from conventional methods.

Based on a Power-to-X concept we calculated the gross electricity demand for the potential production of carbon-based fuels, plastics, and food with the following assumptions: electrochemical reduction of CO_2_ to formate is taken as the primary step, assuming an electrical energy demand of 3.40 MWh/t formate^60,61^. Further reduction of carbon in the cellular metabolism is accounted for by partial oxidation of formate to provide reduction equivalents on a stoichiometric basis. Any losses or process energy demand was not considered for these gross calculations. Projecting electricity costs is apparently the most variable part of this calculation and was allocated at three levels: (i) minimal additional electricity costs of 0.025 €/kWh assumed for peak “surplus” electricity^62^; (ii) photovoltaic electricity costs in 2021 of about 0.058 €/kWh^63^, (iii) the world average price for electricity for industry by end of 2022 of 0.18 €/kWh^64^.

### Fuels

The transportation sector is responsible for 20% of anthropogenic CO_2_ emissions and thus greatly contributes to the climate crisis. Of this cars and buses account for 45.1%, whereas 29.4% of transportation-derived CO_2_ comes from trucks. Aviation and shipping comprise only approximately 10% each, and the lowest emissions are produced by rail travel - accounting only for 1% of transport emissions^65^. Currently, the transportation sector is heavily dependent on fossil fuels, as mostly petrol, diesel, and aviation fuels (kerosene) are produced from petroleum resources^65^. Given the unsustainable nature of fossil fuel supplies, it is both ecologically and economically inevitable to search for more sustainable alternatives.

Significant technological progress in electric vehicles presents a feasible solution to cut emissions from passenger vehicles^66,67^. Additionally, the expansion of electricity generation enables wider use of electric vehicles in cities and rural areas. Biotechnology offers some sustainable routes that can be exploited in this sector as well: Production of high-density liquid transport fuels, also known as biofuels, is technically feasible. Biofuels are produced from plant biomass by microbial conversion^24,44,68–70^. Ethanol and biodiesel are the two forms of biofuels most frequently used today. However, this often includes first-generation feedstocks, such as corn or sugarcane, meaning that biofuels are produced on cropland which could be used for food and feed production. In addition, studies showed that first-generation biofuels significantly decrease biodiversity and food security^71^. Hence, scientists are continuing to develop technologies that allow the conversion of organic waste, agricultural residues (cellulose, hemicellulose), and algae into ‘second’ and ‘third-generation biofuels’ respectively, to provide a more sustainable solution^7,41,68,72^.

The direct conversion of CO_2_ into fuels gained a lot of momentum in research as well. Chemical and biological conversion of CO_2_ into fuels has been demonstrated in many studies^73^. Catalytic conversion of CO_2_ to jet fuel by inexpensive iron-based catalysis was shown to be technologically feasible^74^. Photosynthetic production of fuels like e.g. lipids accumulated in microalgae^69^ offer an alternative to chemical CO_2_ reduction, and microbial routes reducing and condensing carbon from C1 feedstocks are feasible, using metabolic pathways that normally convert sugar-based carbon sources^68^. Generally, fuels will get burned again thereby releasing CO_2_ and only delaying the carbon’s journey to the atmosphere minimally. CO_2_-derived fuels can mitigate CO_2_ emissions as CO_2_ from the air is used to generate fuel and then re-emitted into the environment after burning. Hence, the recycling of carbon in the form of CO_2_ offers an opportunity to produce fuels more sustainably. Notably, the electrical energy demand for C-neutral fuel production is high and the intrinsic inefficiency of combustion engines must be considered which allows only for less than one-third of the fuel energy content to be converted to mechanical energy. In contrast, electric engines convert nearly 80% of the energy to power at the wheels of motor vehicles^75,76^. A hypothetical case where all aviation fuels would be produced with biotechnology via C1 feedstocks would bear the potential to save 1 Gt CO_2_ emissions per year, requiring however electricity in a range of 10,000 TWh which equals one-third of the current annual global electricity production. This huge electricity demand renders C1 based fuel production unfeasible in a foreseeable future, even more when we consider that all this electrical power would need to derive from CO_2_ neutral sources.

Hence, we envisage a future in which the automobile fleet consists mainly of electric vehicles wherever possible. Sectors that are more difficult to electrify, such as aviation or ships can rely on C1-derived fuels or ‘second’ and ‘third-generation biofuels. We do not consider ‘first-generation biofuels’ as a sustainable option as they have several environmental and socioeconomic impacts^77^. While progress in catalyst technology will soon allow commercialization of CO_2_-derived fuels they will not be able to overcome the physical limitations of combustion engines.

### Materials

Around 2020, the total human-made mass of materials exceeded the sum of living biomass on Earth with about 1.1 teratons dry mass^78^. Most of this anthropogenic mass is construction materials (concrete, gravel, bricks, asphalt) and responsible for 4 gigatons of annual CO_2_ emissions^79^. Among the total anthropogenic materials plastics are a comparatively small but rapidly growing fraction with an annual production of 390 megatons in 2021^80^. It was estimated that until 2015, about 8 gigatons of plastics have been produced^81^ which would sum up to approximately 11 gigatons to date.

In general, plastics are a variety of synthetic or semi-synthetic polymers. 99% of the raw materials for plastic production are derived from fossil resources such as mineral oil, gas, or coal, which significantly increase their carbon footprint^82^. 4% of global greenhouse gas emissions are caused solely by the production of plastics^83^, and as plastic production will continuously increase in the future also the carbon footprint will get bigger, not even considering CO_2_ release by decomposition or incineration. Conventional polymers like polyethylene (PE) and polypropylene (PP) have a carbon content of about 85% (mass carbon per total mass) and thus form a potential carbon sink. Considering this carbon content, 300 megatons of carbon can be potentially stored in the annually produced plastics, provided the material is used over a longer time and properly recycled. The biobased plastic polylactic acid (PLA) with a carbon content of 50% offers a theoretical carbon storage potential of up to 200 megatons per year. Using CO_2_ and CO_2_-derived carbon sources would render the production of plastics CO_2_ negative, constituting a potential CO_2_ sink of several 100 megatons per year, given the numbers summarized above.

Microbial production of different precursors for polymers^50,84,85^ or natural microbial polymers such as polyhydroxyalkanoates (PHA)^26,86^ from C1 substrates has been demonstrated. These data show that the conversion of CO_2_ or CO_2_-derived C1 feedstocks into biobased plastics is technically feasible. But would it also be economically feasible? Providing the carbon and energy feedstock for microbial production of lactic acid from CO_2_-derived formate, as an example, replacing all plastics produced to date, would require appr. 5000 TWh per year (based on the calculation that the reduction of CO_2_ to formate demands 0.16 kWh per mol carbon^61^). This would account for energy input costs in the range of 0.78 − 2.31 € per kg PLA, based on solar photovoltaic energy^63^ or average electricity prices for the industry at the end of 2022^64^, respectively. A minimum electricity cost scenario of 0.025 €/kWh would lead to energy input costs of 0.33 €/kg PLA^62^. In comparison, the price for sugar as a feedstock for traditional PLA production ranges around 0.37 €/kg^87^. This scenario illustrates that CO_2_-based biotech processes to produce materials are in principle economically feasible, however just at the edge of competitiveness given the current raw material costs (not accounting for transition costs to new technologies).

Among the largest fractions of construction material, cement and asphalt are major emitters of CO_2_, where cement and concrete production amounts to 8% of the global anthropogenic CO_2_ emissions^88^. While a total replacement of construction materials by biobased products is likely not feasible, we envisage the implementation of e.g., biobased binders in the workflows of the construction industries. Besides, cement production and asphalt paving would make valuable sources of concentrated CO_2_ to be captured and utilized in C1-based production processes.

### Food and feed

Traditional agriculture which developed 12,000 years ago ensured continuous population growth and shaped our civilization tremendously^89^. However, conventional ways of feeding the world have taken a huge burden on our planet; overfishing endangers our seas, cultivation of arable land harms ecosystems and accelerates deforestation, and the climate crisis affects food security in many nations^90^. Extreme weather events like droughts, floods, and storms as well as the prevalence of pests and diseases will additionally burden food security. Currently, food production is accountable for 25–30% of annual CO_2_ emissions, thereby constituting a major driver of the climate crisis^91^. Additionally, we are using 38% of the global land surface for agriculture, of which approximately two-thirds are used as pasture land and one-third as crop land^10,92^. As the global population continues to grow and is expected to exceed 10.4 billion in 2050^93^, the demand for food is growing - and so is the demand for land, a finite resource. Livestock husbandry, which satisfies our need for nutritional protein, contributes significantly to agricultural CO_2_ emissions. In fact, worldwide meat production has exceeded 350 million tons per year and is accountable together with dairy production for 14.5% of annual greenhouse gas emissions, while delivering only 18% of the daily calorie intake consumed by humans^94,95^. Producing and consuming meat, dairy, and other protein products in a way that has less of an impact on the environment is one of the most urgent global concerns.

Microbial protein (MP) offers a sustainable solution when compared to meat or plant-derived proteins. In this perspective, we define MP as cellular protein produced by microbes, such as bacteria, archaea, filamentous fungi, yeasts, or microalgae. Depending on the used microbial platform the content of microbial biomass can vary in lipid, amino acid, and vitamin content, however, the general protein content in MP is quite high, ranging between 30–60% of dry weight^96^. Also, a wide source of carbon substrates can be used, depending on the host system. Methylotrophs, methanotrophs, or acetotrophs offer a platform for biomass production on C1-derived methanol, formate, methane, or acetic acid. Autotrophs can convert CO_2_ directly into biomass. Thus, MP production offers great flexibility in process design, and using CO_2_ as a carbon source would render the process C-neutral. Many studies demonstrated that the production of MP significantly reduces the amount of land and water used^97–99^. Just to mention a few examples, Humpenöder et al. ^100^ showed that the annual deforestation and associated CO_2_ emissions could be reduced by half if fungi were used to replace 20% of cow meat by 2050. Also, Pikaar et al. ^101^ demonstrated that MPs can reduce farmland area, nitrogen losses, and greenhouse emissions by 6, 8 and 7% respectively, if only 10–19% of typical crop-based animal feed protein is replaced. And theoretically according to Dorian Leger et al. ^99^, photovoltaic-driven MP (PV-MP) production could outperform conventional staple crops in terms of both calorie and protein production. In detail, PV- MP production may deliver a 10-fold increase in protein and 2-fold increase in caloric yield when compared to conventional staple crops. They also claim that PV-MP production can surpass MP production driven by first-generation feedstocks, hence bearing enormous potential for a land and energy efficient alternative. Noteworthy, H_2_ and methanol in combination with the CBB and RuMP cycle, respectively, are proposed to deliver the highest energetic efficiencies. Thus, with smart process design and the utilization of C1-derived carbon sources, MP production has great potential to mitigate CO_2_ emissions and save precious land and water resources. It should be noted that total microbial biomass contains approximately 5–15% nucleic acid which needs to be reduced for food application as they can lead to increased uric acid levels causing gout and kidney stones. Additional processing like RNAse treatment or alkaline extraction leads to loss of dry mass and may also cause additional CO_2_ emissions^96^.

Another strategy to replace animal proteins for human nutrition is through recombinant protein production via fermentation. Recombinant protein production enables the synthesis of bio-identical animal proteins like milk casein, whey, or egg ovalbumin without the usage of animals^102^. All types of microorganisms, such as the bacterium *E. coli*, the yeast *K. phaffii*, or the filamentous fungus *Rhizopus*, have been engineered to produce milk proteins recombinantly^103^. Recombinant production of milk protein is technologically feasible, however low yields and divergent posttranslational modifications of the proteins, including glycosylation, or the additional secretion of proteases still challenge the commercialization of final products^104^. Similarly, hen egg ovalbumin is being produced in *Trichoderma reesei*^105^ and *K. phaffii*^106^. Start-ups like Every Company, Perfect Day, New Culture, Formo, and Those Vegan Cowboys are advancing recent research in this area^104^. Although more research is needed to solve some of the aforementioned issues, vegan dairy or egg products made of animal-free proteins produced by microbes are a promising sustainable alternative to conventional products. Additionally, further genetic engineering or exploitation of C1-utilizing microorganisms can improve the ecological footprint by establishing C-neutral production processes^107,108^.

## Concluding remarks

The scope and complexity of the multifaceted challenges inflicted by the climate crisis that must be addressed are daunting. The fuel, material, and food sectors are three major CO_2_ emitters. In this Perspective, we have elaborated on implementing C1 feedstocks in the aforementioned sectors to create more sustainable production cycles. For that, natural or synthetic pathways for C1 utilization can be exploited, and as progress in research shows, the potential of synthetic biology is immense to explore and modify metabolic pathways for efficiency and product variety. In terms of fuel efficiency, electric engines are the most efficient; hence, replacing fossil fuels with C1-derived fuels makes more sense when liquid fuels are unavoidable. According to our estimations, replacing aviation fuel with C1-derived fuels would need an additional 30% of the world’s electricity demand. Contrary to the fuel sector, the implementation of C1 substrates in the material sector is more straightforward. Advancements in metabolic engineering, synthetic biology, and industrial process design enabled microbes to utilize these next-generation feedstocks. Microbial polymer synthesis from C1 substrates, in particular, has the potential to build a carbon sink and reduce CO_2_ emissions. Our calculations also underline their economic feasibility. Additionally, by substituting first-generation feedstocks, land, and water resources can be saved. The scenario for the food and feed sector looks very similar. Partial replacement of conventional food and feed with either MP or specific animal-free proteins such as caseins, whey, and egg ovalbumin pave the way for more sustainable processes, with less impact on water and land resources, biodiversity, and animal suffering. However, implementing C1 feedstocks into processes requires more than scientific and technological advances. Although replacing all important industries with C1 carbon sources is appealing, a sustainable future requires also realistic and practical solutions. This does not mean we do not aim high, but all aspects should be considered when proposing implementation of C1-feedstocks with conventional processes. We should consider whether industrial processes or industries may have an influence on CO_2_ levels when combined with C1 feedstocks, and to which degree this helps us reduce CO_2_ emissions and combat the climate crisis. On top, we should rethink production and consumption cycles for their environmental impact. E.g., a reduction of annual plastics production and turnover would have multiple benefits beyond CO_2_ neutral production, same as a shift from individual traffic to public transportation. In addition, the benefits of C1 feedstocks for society and the global economy may not be realized if public acceptability is not appropriately addressed. Public responses can be crucial for the success of technologies, and from the past, we know that unacceptance can lead to failure as this was the case for genetically modified foods in Europe^109^. We also anticipate that industries, together with engineers, scientists, and policymakers, should enable innovative bioprocesses that contribute to a sustainable economy, as immediate action is required.

### Supplementary information

Supplementary Information
