# Supplementary material for: The potential of CO2-based production cycles in biotechnology to fight the climate crisis
Source: Nat Commun. 2023 Nov 1;14:6978. doi: 10.1038/s41467-023-42790-6 (PMC10620168; doi:10.1038/s41467-023-42790-6)
Supplement: Supplementary file 1 — Supplementary Information [file 41467_2023_42790_MOESM1_ESM.pdf]

The potential of CO_2-based production cycles in biotechnology to fight the climate crisis

Bachleitner *et al.*

Supplementary Table 1. Calvin-Benson-Bassham (CBB) cycle reactions used for the calculation of the minimal metabolic driving force by eQuilibrator.

**Reaction**	**Relative flux**
CO2 + ribulose 1,5-bisphosphate + H2O <=> 2 3-phospho d-glycerate	3
ATP + 3-phospho d-glycerate <=> ADP + Glycerate-1,3-Bisphosphate	6
Glycerate-1,3-Bisphosphate + nadph <=> D-glyceraldehyde 3-phosphate + Pi + nadp	6
d-glyceraldehyde 3-phosphate <=> glycerone phosphate	2
Glycerone phosphate + D-Glyceraldehyde 3-phosphate <=> D-fructose 1,6-bisphosphate	1
D-fructose 1,6-bisphosphate + H2O <=> D-fructose 6-phosphate + Pi	1
D-fructose 6-phosphate + D-glyceraldehyde 3-phosphate <=> xylulose 5-phosphate + erythrose 4-phosphate	1
Erythrose 4-phosphate + glycerone phosphate <=> Sedoheptulose 1,7-bisphosphate	1
Sedoheptulose 1,7-bisphosphate + H2O <=> sedoheptulose 7-phosphate + Pi	1
sedoheptulose 7-phosphate + D-glyceraldehyde 3-phosphate <=> ribose 5-phosphate + xylulose 5-phosphate	1
D-ribose 5-phosphate <=> D-ribulose 5-phosphate	1
D-xylulose 5-phosphate <=> D-ribulose 5-phosphate	2
D-ribulose 5-phosphate + ATP <=> ribulose 1,5-bisphosphate + ADP	3

Supplementary Table 2. Reductive tricarboxylic acid cycle (rTCA) reactions used for the calculation of the minimal metabolic driving force by eQuilibrator.

**Reaction**	**Relative flux**
oxaloacetate + NADH <=> malate + NAD+	1
malate <=> fumarate + H2O	1
fumarate + FADH2 <=> succinate + FAD	1
succinate + Coenzyme A + ATP <=> succinyl-CoA + ADP + Pi	1
succinyl-CoA + CO2 + NADH <=> alpha-ketoglutarate + Coenzyme A + NAD+	1
alpha-ketoglutarate + CO2 + NADH <=> isocitrate + NAD+	1
Isocitrate <=> cis-aconitate + H2O	1
cis-aconitate + H2O <=> Citrate	1
citrate + ATP + Coenzyme A <=> AcetylCoA + oxaloacetate + ADP + Pi	1

Supplementary Table 3. Wood Ljungdahl pathway reactions used for the calculation of the minimal metabolic driving force by eQuilibrator.

Reaction	Relative flux
CO2 + NADPH <=> Formate + NADP+	1
Tetrahydrofolate + Formate + ATP <=> ADP + Pi + 10-Formyltetrahydrofolate	1
10-Formyltetrahydrofolate <=> 5,10-Methenyltetrahydrofolate + H2O	1
5,10-Methenyltetrahydrofolate + NADPH <=> 5,10-Methylenetetrahydrofolate + NADP+	1
5,10-Methylenetetrahydrofolate + NADH <=> 5-Methyltetrahydrofolate + NAD+	1
CO2 + H2 <=> Carbon monoxide + H2O	1
5-methyltetrahydrofolate + Coenzyme A + Carbon monoxide <=> Acetyl-CoA + Tetrahydrofolate	1
H2 + NAD+ <=> 2H+ + NADH	1
H2 + NADP+ <=> 2H+ + NADPH	2

Supplementary Table 4. Reductive glycine (rGlycine) pathway reactions used for the calculation of the minimal metabolic driving force by eQuilibrator.

Reaction	Relative flux
Tetrahydrofolate + Formate + ATP <=> ADP + Pi + 10-Formyltetrahydrofolate	2
10-Formyltetrahydrofolate <=> 5,10-Methenyltetrahydrofolate + H2O	2
5,10-Methenyltetrahydrofolate + NADPH <=> 5,10-Methylenetetrahydrofolate + NADP+	2
5,10-Methylenetetrahydrofolate + NH3 + CO2 + NADH <=> Glycine + Tetrahydrofolate + NAD+	1
Glycine + 5,10-Methylenetetrahydrofolate + H2O <=> serine + tetrahydrofolate	1
serine <=> pyruvate + NH3	1

Supplementary Table 5. Serine cycle reactions used for the calculation of the minimal metabolic driving force by eQuilibrator.

**Reaction**	**Relative flux**
Methanol + Ubiquinone <=> Formaldehyde + Ubiquinol	1
Formaldehyde + H2O + NAD+ <=> Formate + NADH	1
Tetrahydrofolate + Formate + ATP <=> ADP + Pi + 10-Formyltetrahydrofolate	1
10-Formyltetrahydrofolate <=> 5,10-Methenyltetrahydrofolate + H2O	1
5,10-Methenyltetrahydrofolate + NADPH <=> 5,10-Methylenetetrahydrofolate + NADP+	1
5,10-Methylenetetrahydrofolate + Glycine + H2O <=> Tetrahydrofolate + L-Serine	1
L-Serine + Glyoxylate <=> Hydroxypyruvate + Glycine	1
Hydroxypyruvate + NADH <=> Glycerate + NAD+	1
Glycerate + ATP <=> 3-Phosphoglycerate + ADP	1
3-Phosphoglycerate <=> Phosphoenolpyruvate + H2O	1
Phosphoenolpyruvate + Carbon dioxide + H2O <=> Oxaloacetate + Pi	1
Oxaloacetate + NADH <=> Malate + NAD+	1
ATP + Malate + Coenzyme A <=> ADP + Pi + Malyl-CoA	1
Malyl-CoA <=> Acetyl-CoA + Glyoxylate	1

Supplementary Table 6. Xylulose monophosphate (XuMP) cycle reactions used for the calculation of the minimal metabolic driving force by eQuilibrator.

Reaction	Relative flux
methanol + O2 <=> formaldehyde + H2O2	3
formaldehyde + D-xylulose 5-phosphate <=> Glycerone + D-Glyceraldehyde 3-phosphate	3
glycerone + ATP <=> glycerone phosphate + ADP	3
Glycerone phosphate + D-Glyceraldehyde 3-phosphate <=> d-fructose 1,6-bisphosphate	1
D-fructose 1,6-bisphosphate + H2O <=> D-fructose 6-phosphate + Pi	1
D-fructose 6-phosphate + D-glyceraldehyde 3-phosphate <=> xylulose 5-phosphate + erythrose 4-phosphate	1
Erythrose 4-phosphate + glycerone phosphate <=> Sedoheptulose 1,7-bisphosphate	1
Sedoheptulose 1,7-bisphosphate + H2O <=> sedoheptulose 7-phosphate + Pi	1
sedoheptulose 7-phosphate + D-glyceraldehyde 3-phosphate <=> ribose 5-phosphate + xylulose 5-phosphate	1
ribose 5-phosphate <=> xylulose 5-phosphate	1
Glycerone phosphate <=> D-Glyceraldehyde 3-phosphate	1

Supplementary Table 7. Xylulose monophosphate (XuMP) cycle reactions without sedoheptulose-1,7-bisphosphatase (SBP) to calculate the minimal metabolic driving force by eQuilibrator.

**Reaction**	**Relative flux**
methanol + O2 <=> formaldehyde + H2O2	3
formaldehyde + D-xylulose 5-phosphate <=> Glycerone + D-Glyceraldehyde 3-phosphate	3
glycerone + ATP <=> glycerone phosphate + ADP	3
Glycerone phosphate + D-Glyceraldehyde 3-phosphate <=> d-fructose 1,6-bisphosphate	2
D-fructose 1,6-bisphosphate + H2O <=> D-fructose 6-phosphate + Pi	2
D-fructose 6-phosphate + D-glyceraldehyde 3-phosphate <=> xylulose 5-phosphate + erythrose 4-phosphate	1
D-fructose 6-phosphate + erythrose 4-phosphate <=> sedoheptulose 7-phosphate + D-glyceraldehyde 3-phosphate	1
sedoheptulose 7-phosphate + D-glyceraldehyde 3-phosphate <=> ribose 5-phosphate + xylulose 5-phosphate	1
ribose 5-phosphate <=> xylulose 5-phosphate	1
Glycerone phosphate <=> D-Glyceraldehyde 3-phosphate	1

Supplementary Table 8. Ribulose monophosphate (RuMP) cycle (transaldolase variant, TA) reactions to calculate the minimal metabolic driving force by eQuilibrator.

**Reaction**	**Relative flux**
methanol + O2 <=> formaldehyde + H2O2	3
D-ribulose 5-phosphate + formaldehyde <=> hexulose 6-phosphate	3
hexulose 6-phosphate <=> D-fructose 6-phosphate	3
ATP + D-fructose 6-phosphate <=> ADP + D-fructose 1,6-bisphosphate	1
D-fructose 6-phosphate + D-glyceraldehyde 3-phosphate <=> D-xylulose 5-phosphate + D-erythrose 4-phosphate	1
D-fructose 1,6-bisphosphate <=> glycerone phosphate + D-glyceraldehyde 3-phosphate	1
glycerone phosphate <=> D-glyceraldehyde 3-phosphate	1
D-erythrose 4-phosphate + D-fructose 6-phosphate <=> D-sedoheptulose 7-phosphate + D-glyceraldehyde 3-phosphate	1
D-sedoheptulose 7-phosphate + D-glyceraldehyde 3-phosphate <=> D-ribose 5-phosphate + D-xylulose 5-phosphate	1
D-ribose 5-phosphate <=> D-ribulose 5-phosphate	1
D-xylulose 5-phosphate <=> D-ribulose 5-phosphate	2

Supplementary Table 9. Ribulose monophosphate (RuMP) cycle (sedoheptulose-1,7-bisphosphatase variant, SBP) reactions to calculate the minimal metabolic driving force by eQuilibrator.

**Reaction**	**Relative flux**
methanol + O2 <=> formaldehyde + H2O2	3
D-ribulose 5-phosphate + formaldehyde <=> hexulose 6-phosphate	3
hexulose 6-phosphate <=> D-fructose 6-phosphate	3
ATP + D-fructose 6-phosphate <=> ADP + D-fructose 1,6-bisphosphate	2
D-fructose 6-phosphate + D-glyceraldehyde 3-phosphate <=> D-xylulose 5-phosphate + D-erythrose 4-phosphate	1
D-fructose 1,6-bisphosphate <=> glycerone phosphate + D-glyceraldehyde 3-phosphate	2
glycerone phosphate <=> D-glyceraldehyde 3-phosphate	1
glycerone phosphate + D-erythrose 4-phosphate <=> D-sedoheptulose 1,7-bisphosphate	1
D-sedoheptulose 1,7-bisphosphate + H2O <=> D-sedoheptulose 7-phosphate + Pi	1
D-sedoheptulose 7-phosphate + D-glyceraldehyde 3-phosphate <=> D-ribose 5-phosphate + D-xylulose 5-phosphate	1
D-ribose 5-phosphate <=> D-ribulose 5-phosphate	1
D-xylulose 5-phosphate <=> D-ribulose 5-phosphate	2

Supplementary Table 10. Carbon fixing pathways and their energy demand when pyruvate is the output molecule for each pathway. For the conversion of Acetyl-CoA to pyruvate, a reaction catalyzed by pyruvate ferredoxin oxidoreductase with NADH as reducing equivalent was added.

Pathway	Net reaction	Input	Output	ATP/ C-mol	NAD(P)H/ C-mol
**CBB cycle**	4 H_2O + 7 ATP + 5 NADPH + 3 CO2 <=> 5 NADP + 7 ADP + 7 phosphate + pyruvate	CO_2	Pyruvate	2.33	1.67
**rTCA cycle**	2 ATP + 4 NADH + 3 CO_2 + $FADH_2$ <=> H2O + 2 ADP + 4 NAD + 2 phosphate + pyruvate + FAD	CO_2	Pyruvate	0.67	1.33 (+0.33 FADH)
**Wood Ljungdahl pathway**	ATP + NADH + 3 CO_2 + 4 H_2 <=> 2 H_2O + ADP + NAD + phosphate + pyruvate	CO_2	Pyruvate	0.33	1.67
**rGlycine pathway**	2 ATP + 2 NADPH + NADH +CO_2 + 2 formate <=> H_2O + 2 NADP + 2 ADP + NAD + 2 phosphate + pyruvate	Formate, CO_2	Pyruvate	0.67	1
**Serine cycle**	H_2O + 3 ATP + NADPH + 2 NADH + 2 CO_2 + methanol + ubiquinone <=> NADP + 3 ADP + 2 NAD + 3 phosphate + pyruvate + ubiquinol	Methanol, CO_2	Pyruvate	1	1
**XuMP cycle**	H_2O + ATP + 3 O_2 + NAD + 3 methanol <=> ADP + phosphate + NADH + 3 H_2O_2 + pyruvate	Methanol	Pyruvate	0.33	- 0.33
**XuMP cycle w/o SBP**	H_2O + ATP + 3 O_2 + NAD + 3 methanol <=> ADP + phosphate + NADH + 3 H_2O_2 + pyruvate	Methanol	Pyruvate	0.33	- 0.33
**RuMP cycle TA version**	3 O_2 + ADP + NAD + phosphate + 3 methanol <=> H_2O + ATP + NADH + 3 H_2O_2 + pyruvate	Methanol	Pyruvate	- 0.33	- 0.33
**RuMP cycle SBP version**	3 O_2 + NAD + 3 methanol <=> NADH + 3 H_2O_2 + pyruvate	Methanol	Pyruvate	0	- 0.33
